# Extracellular Vesicles as Novel Diagnostic and Prognostic Biomarkers for Parkinson’s Disease

**DOI:** 10.14336/AD.2021.0527

**Published:** 2021-09-01

**Authors:** Loredana Leggio, Greta Paternò, Silvia Vivarelli, Giovanna G Falzone, Carmela Giachino, Bianca Marchetti, Nunzio Iraci

**Affiliations:** ^1^Department of Biomedical and Biotechnological Sciences (BIOMETEC), University of Catania, Torre Biologica, 95125 Catania, Italy.; ^2^Neuropharmacology Section, OASI Research Institute-IRCCS, 94018 Troina, Italy.

**Keywords:** Biomarkers, Extracellular Vesicles, Exosomes, Mitochondria-Derived Vesicles (MDVs), Parkinson’s disease, miRNA

## Abstract

The elderly population will significantly increase in the next decade and, with it, the proportion of people affected by age-related diseases. Among them, one of the most invalidating is Parkinson’s disease (PD), characterized by motor- and non-motor dysfunctions which strongly impair the quality of life of affected individuals. PD is characterized by the progressive degeneration of dopaminergic neurons, with consequent dopamine depletion, and the accumulation of misfolded α-synuclein aggregates. Although 150 years have passed since PD first description, no effective therapies are currently available, but only palliative treatments. Importantly, PD is often diagnosed when the neuronal loss is elevated, making difficult any therapeutic intervention. In this context, two key challenges remain unanswered: (i) the early diagnosis to avoid the insurgence of irreversible symptoms; and (ii) the reliable monitoring of therapy efficacy. Research strives to identify novel biomarkers for PD diagnosis, prognosis, and therapeutic follow-up. One of the most promising sources of biomarkers is represented by extracellular vesicles (EVs), a heterogeneous population of nanoparticles, released by all cells in the microenvironment. Brain-derived EVs are able to cross the blood-brain barrier, protecting their payload from enzymatic degradation, and are easily recovered from biofluids. Interestingly, EV content is strongly influenced by the specific pathophysiological status of the donor cell. In this manuscript, the role of EVs as source of novel PD biomarkers is discussed, providing all recent findings concerning relevant proteins and miRNAs carried by PD patient-derived EVs, from several biological specimens. Moreover, the contribution of mitochondria-derived EVs will be dissected. Finally, the promising possibility to use EVs as source of markers to monitor PD therapy efficacy will be also examined. In the future, larger cohort studies will help to validate these EV-associated candidates, that might be effectively used as non-invasive and robust source of biomarkers for PD.

Review

## 1. Introduction

### 1.1 Parkinson’s disease: an aging related disorder

Aging is a universal but not uniform process, characterized by multiple and complex mechanisms (e.g., genomic instability, epigenetic alterations, telomere shortening, deregulated nutrient sensing, mitochondrial dysfunction, stem cell exhaustion, alteration in intercellular signaling etc.) [1]. Aging is recognized as a major risk factor for neurodegenerative diseases (NDs), and particularly Parkinson’s disease (PD).

PD is the most prevalent central nervous system (CNS) movement disorder affecting about 7 million people worldwide over the age of 65 [2, 3]. PD is also one of the fastest growing ND, and with the predicted increase in lifespan of the population, a further rise is expected to occur on a yearly basis [4]. At the histopathological level, (i) the selective degeneration of dopaminergic (DAergic) neurons of the substantia nigra (SN) and their terminals in the striatum (Str); and (ii) the presence of intracellular aggregated inclusions containing α-synuclein (α-Syn), called Lewy bodies (LB), represent major hallmarks of the disease [5-7]. Additionally, an abnormal activation of the glial cell compartment precedes and/or accompany PD development and progression [8-13]. As a resulting feature of nigral degeneration, dopamine (DA) storage in the Str progressively decreases to a critical threshold level, when the typical clinical signs (namely, bradykinesia, tremor, and rigidity) finally manifest [5, 14, 15].

Unfortunately, by that time, up to 50% of DAergic cell bodies and close to 80% of Str DAergic terminals are already lost, and even if the progression of the disease is slow in most cases, the nigrostriatal DAergic degeneration is irreversible. Along with the motor signs, a number of non-motor symptoms, including autonomic, sleep, cognitive, and mental health disorders, typically precede PD onset and progression, with mechanisms not completely understood [15-18]. The preclinical, latent, so-called prodromic phase, estimated to last 8-17 years, has suggested the existence of compensatory mechanisms likely responsible for the gradual adaptation of the DA neuronal system [19-21]. This stage of the disorder is of particular relevance, in relation to the development of disease-modifying or neuroprotective therapies which would require intervention at the earliest stages of disease [22].

However, current therapies for PD are still mainly directed towards replacing DA levels in the brain, with (i) L-3,4-dihydroxyphenylalanine (L-DOPA, the precursor of DA); (ii) inhibitors of DA metabolism; (iii) DA receptor agonists; and (iv) by increasing DA release [15]. Of note, while providing a symptomatic relief [15, 23], these drugs do not modify the progressive neurodegenerative cell loss associated with PD, and in many cases, they result in debilitating side-effects [15]. Indeed, despite the significant scientific advancement of the last decades, the causes, mechanisms and determinant factors leading to DAergic neuron demise in PD still remain poorly understood [24]. Over the years, numerous novel therapies are being suggested along with development of medications to treat various non-motor symptoms to improve quality of life of patients, and excellent reviews have recently discussed these topics elsewhere [15, 25-27].

Aside the early-onset familial PD, most (≥ 90%) PD cases are sporadic, with current evidence indicating a complex interplay between genetic susceptibility and a panel of environmental factors contributing to PD pathophysiology [3, 12, 28-36]. Indeed, several genes - including α-Syn (SNCA), parkin (PRKN), PTEN-induced putative kinase (PINK1), protein deglycase (DJ-1), and leucine-rich-repeat kinase 2 (LRRK2) - and many environmental factors - chiefly aging and inflammation - impact on the regulation of crucial pathways involved in oxidative stress, mitochondrial function, endoplasmic reticulum stress, autophagic catabolism, protein misfolding and aggregation, finally resulting in the progressive loss of DAergic neurons [28, 34, 37-42].

Due to the important structural and functional changes occurring both at the CNS and periphery [43], aging represents a critical phase of life for PD development and progression [44]. Especially, oxidative stress and low-grade inflammation are considered crucial hallmarks of aging. Both processes are upregulated upon injury, as well as upon a panel of genetic and environmental factors [45-48]. In fact, with the aging process, in brain glial cell compartment, astrocytes and microglia lose their neuroprotective potential, and become more responsive to various stimuli, thus producing higher levels of inflammatory and oxidative stress mediators, such as tumor necrosis factor α (TNF-α), interleukin 1β (IL-1β) and IL-6 [49, 50].

Of significance, with age and environmental toxic exposures, glial cells increasingly display a senescence-associated secretory phenotype, further contributing to drive aging and loss of tissue homeostasis, in turn contributing to PD neuropathology [51]. Additionally, both the genetic background and the exposure to inflammatory challenges can promote a self-perpetuating cycle of microglial-mediated DAergic neurotoxicity, in the context of a dysfunctional astrocyte-microglia cross-talk [28]. The resulting inflammatory microenvironment is associated with oxidative stress mediators, such as reactive oxygen (ROS) and nitrogen species (RNS), that in turn amplify microglial activation with detrimental effects for mitochondrial homeostasis, finally leading to increased DAergic neuron vulnerability, and/or neuronal death [52-57]. Altogether, both aging and inflammation dramatically impact on DAergic neuron vulnerability, as well as on the capacity for neurorepair in PD, as a result of a vicious crosstalk between genetic and environmental factors. In this context, extracellular vesicles (EVs) are key players in glial-neuron dialogue during both the development and/or the progression of the disease (see section 1.2).

Because slowing down or halting PD still remains the principal goal of the research [15, 58], a major challenge regards the possibility to uncover exactly when and how the first signs of the disease do appear, in order to develop therapeutic strategies to mitigate or stop the progressive DAergic neuronal death. To this end, considerable effort is devoted to the screening of valuable clinical PD biomarkers, to reveal early aspects of disease pathogenesis and eventually to develop diagnostic methods to prevent and treat PD [59-63]. Investigations aimed at identifying preclinical PD biomarkers represent an important step towards the development of more efficacious neuroprotective therapies. This would delay the onset and progression of the disease - limiting the ongoing degeneration process before the appearance of the first clinical symptoms - and help to unravel novel etiopathogenetic mechanisms/targets for PD. The possibility to identify early signs of the disease investigating pre-clinical biomarkers, in particular within EVs, is summarized in the following sections.

### 1.2 Extracellular vesicles: key players in cell-to-cell communication

A growing body of research suggests that EVs may represent an important source of novel biomarkers. EVs are lipid nanometric structures potentially released in their surrounding microenvironment by all types of cells [64]. Based on their dimension, EVs are classified in small EVs (< 200 nm) and medium/large EVs (> 200 nm) [65]. Exosomes, small shedding vesicles and mitochondria-derived vesicles (MDVs) belong to the group of small EVs [66]. On the other hand, larger microvesicles, ectosomes, oncosomes are comprised in the category of medium/large EVs. While most of the EVs derive from plasma membrane budding, exosomes possess a specific biogenesis pathway. In fact, they are generated upon intraluminal vesicles formation within the multivesicular body (MVB) compartment. Next, exosomes are released outside the cell following MVB fusion with plasma membrane [67].

EVs might contain almost all categories of biomolecules, such as nucleic acids (i.e. nuclear and mitochondrial DNA, long and small RNAs), proteins (i.e. structural and/or enzymatic proteins), metabolites and lipids [68, 69]. Lipids are the essential component of the EV bilayer membrane. The lipid composition of EVs is dependent on the cell source, and also on the specific EV type. As recently reviewed by Skotland et al, lipids in EVs belong to different classes (e.g., glycerophospholipids, sphingolipids, cholesterol, cholesterol esters, ceramide etc.), with distinct distribution between the inner and outer side of the bilayer [70]. In addition, it has been demonstrated that some classes of lipids (e.g. cholesterol, sphingomyelin, phosphatidylcholine, phosphatidylserine, phosphatidylethanolamine, ceramide, and others) are enriched in EVs as compared to their donor cells [70]. Furthermore, specific EV-associated lipid signatures have been linked with CNS disorders, including PD [71].

Several types of proteins may be also found in EVs, both transmembrane/lipid-anchored and soluble in the vesicle lumen [65]. These include tetraspanins (e.g. CD63, CD9, CD81), adhesion molecules (e.g. L1CAM), integrins, components of the endosomal sorting complex required for transport (ESCRT; e.g., Tsg101), but also receptors (e.g. the interferon γ receptor 1 [72]) and enzymes (e.g. asparaginase-like protein 1 [73]. Regarding EV-membrane proteins, mass spectrometry studies revealed that some of them may have a reverse orientation compared with donor cells, implying a potential alteration of the biological functions in recipient cells [74]. Notably, as mentioned for lipids, also the EV proteome depends on the identity and the physio-/pathological status of the donor cell [75]. The presence of nucleic acids in EVs is similarly diverse, with old and new classes of ncRNAs (e.g., tRNA-derived fragments) being identified as potential functional cargoes (for a detailed review see [76]).

This complexity in the EV composition is reflected by the plethora of their biological functions, which makes EVs key players for cell-to-cell communication in many different contexts, as extensively reviewed elsewhere [77, 78]. Indeed, once released extracellularly, EVs may convey their information either to adjacent or distant cells. EVs can signal to target cells through different mechanisms, such as membrane fusion, by which the vesicle content is released inside the lumen of the target cell. Other mechanisms include the uptake of intact vesicles (via various mechanisms, including macropinocytosis, phagocytosis, endocytosis via caveolae and lipid rafts, clathrin-dependent endocytosis) [79]. Once inside the target cell, intact EVs may be directed towards distinct cellular compartments where their content is differentially processed. Finally, thanks to the presence of surface ligands/receptors, EVs may interact with the corresponding receptor/ligand expressed at the surface of the target cell, thus triggering a specific response within the target cell [72].

In the context of PD, glial cells can deliver EVs carrying various messages for the vulnerable/dysfunctional DAergic neurons, with important consequences for their death/survival [80]. Of special importance, aging may represent a crucial factor influencing the way by which glial-derived EVs can trigger the development and/or the progression of the disease. In fact, glial EV-derived cargoes are susceptible to play important roles, in either a beneficial or harmful way [80]. The aging process alters not only astrocyte- and microglial- EV content, but also their spreading in the microenvironment, thereby directing both pro/anti-inflammatory responses to the target neuronal cells with consequences for neuronal vulnerability, death and/or repair [81]. Recently, the application of EVs as innovative cell-free therapeutics is being extensively explored. For instance, EVs are under evaluation as nanocarriers for several biomolecules of therapeutic relevance in PD, including L-DOPA [82].

Interestingly, EVs are able to cross biological barriers, such as the blood-brain barrier (BBB), reaching the systemic circulation or other biofluids [67, 83]. EVs released from different cell types (e.g., neurons) can be easily recovered from serum, plasma, urine and other body fluids [83]. Given that the EV content is strictly dependent on the status of the donor cells, the messages carried by EVs may vary between physiological or pathological conditions [80]. Starting from the first reports on the EV potential as novel source of biomarkers [84], the progression of omics approaches allowed to extensively analyze the content of EVs isolated from several sources and in different pathological conditions, including PD [85, 86]. The technical considerations about the EV purification protocols have been already discussed elsewhere [87].

Overall, the goal of this review is to outline a broader perspective in support of the use of EVs as a robust source of biomarkers for PD, and, more in general, for other NDs.

## 2. Discovering novel biomarkers for PD

As mentioned earlier, the discovery of novel PD biomarkers may: i) improve data collection; ii) increase the knowledge about the clinical and the pathological parameters of the disease (i.e., molecular mechanisms underlying PD onset); iii) help monitoring PD progression and treatment efficacy. Unfortunately, PD is a multifaceted pathology with clinical symptoms not only typical of PD, but shared with other forms of parkinsonism. Therefore, a given biomarker could be relevant for certain conditions, but not for others. For all these reasons, despite the efforts made by the scientific community, a valid biomarker for monitoring PD onset and/or progression has not been established yet [88].

Potential biomarkers for PD can be grouped into three main categories: i) brain imaging biomarkers (e.g. Magnetic Resonance Imaging - MRI, Computed Tomography - CT, Positron Emission Tomography - PET etc.); ii) clinical testing procedures (e.g. tests of motor performance, vision, olfaction etc.); and iii) biochemical and cellular biomarkers (e.g. blood tests) [89, 90]. Biomarkers from the third group deserve special attention because they are generally more sensitive, less variable, more easily preserved, simpler to measure. Also, changes in their levels may be the result of the first response of the organism to environmental agents or pharmacological drugs [91].

EVs can be considered a useful tool for biomarker discovery for several reasons. First of all, given their intracellular origin, EVs mirror the pathophysiology of the donor cell. Also, the external lipid bilayer protects the internal content of EVs. In particular, brain-derived EVs are able to transport their cargoes through the BBB to the systemic circulation, where they can be isolated and then analyzed [92]. Considering that also peripheral cells can release vesicles in the biologic fluids, several protocols are being developed to isolate EVs from a defined body district. For example, by using the L1CAM antibody it is possible to isolate the vesicular fraction selectively secreted by neural cells, from the whole blood [93].

**Figure 1.**
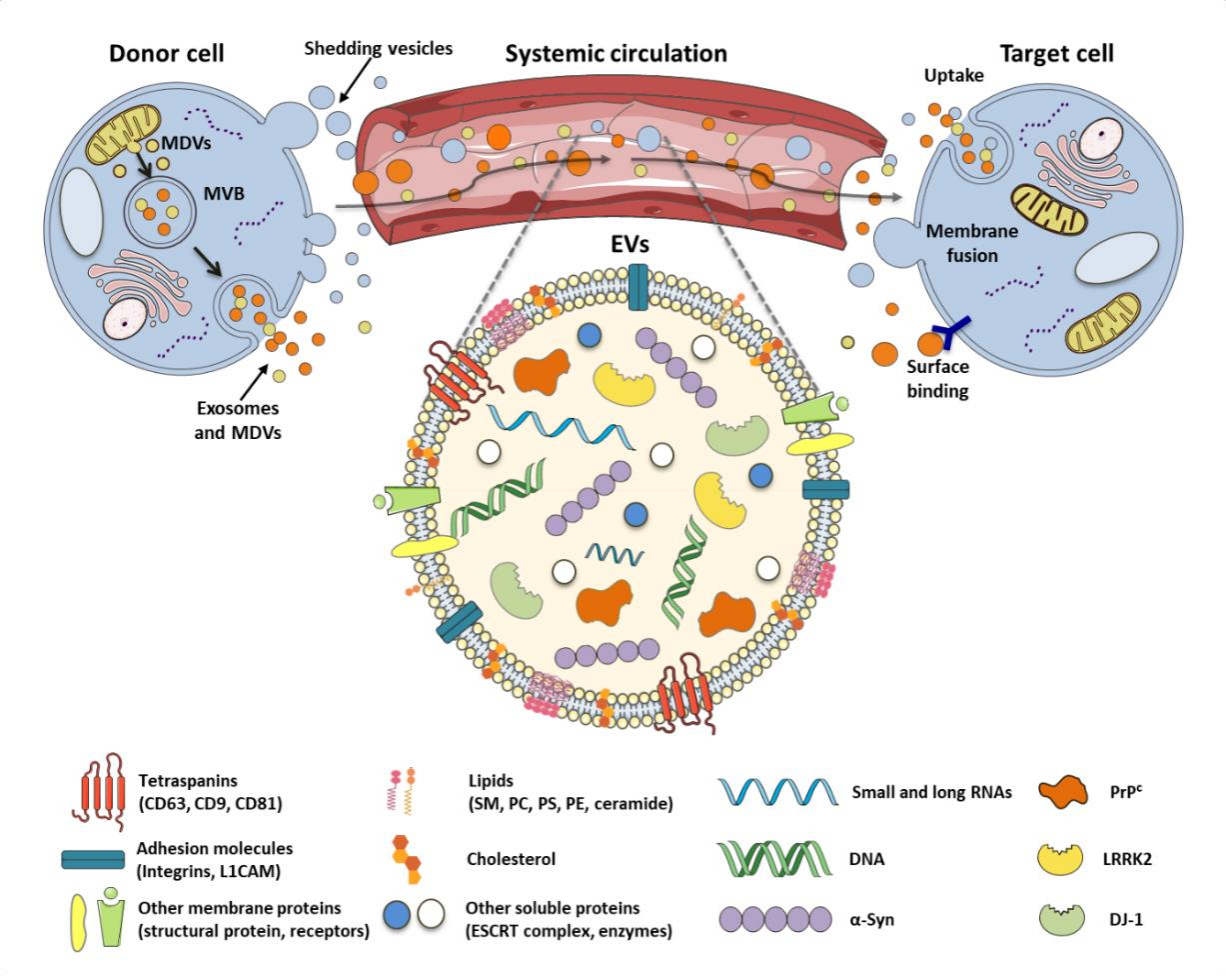
**Potential EV-associated biomarkers for PD**. Exosomes (in orange), MDVs (in yellow) and shedding vesicles (in light blue) are secreted in the microenvironment through different biogenetic pathways: fusion of MVB with the plasma membrane and shedding mechanism. EVs are heterogeneous lipid bilayer structures enriched in sphingomyelin (SM), phosphatidylcholine (PC), phosphatidylethanolamine (PE), phosphatidylserine (PS), ceramide and cholesterol. EVs can carry different cargoes (DNA, miRNAs, Alfa-Synuclein, PrP^C^, LRRK2, DJ-1 and other proteins) from the brain, through the BBB, toward the systemic circulation, where they can be detected as biomarkers. Vesicles reach the target cells through different mechanisms such as uptake or surface binding.

Notably, a growing body of studies demonstrated how the pathological conditions may alter the vesicular content, suggesting therefore that EVs represent a potential diagnostic tool for an early detection of NDs, including PD [94] (See Fig. 1).

In this work we present an up-to-date collection of potential EV-based biomarkers, which include the most important PD-linked proteins and miRNAs. In addition, we introduce a new class of small EVs, of mitochondrial origin (i.e., MDVs), discussing their emerging role as biomarker source in PD. Given the crucial role of aging, inflammation and oxidative stress in PD development and progression, identifying PD-specific EV molecular cargoes that precede and/or accompany the preclinical and clinical phases of the disease would help to assess EV role in PD physiopathology. Along these lines, the prognostic value of EVs is also proposed. Finally, the main challenges related to the application of EVs as biomarkers in the clinical routine are further discussed.

## 3. α-Syn and EVs: a dual role

### 3.1 α-Syn spreading in PD

As well known, the presence of α-Syn aggregates in LB is a typical hallmark of PD. α-Syn is a 140 amino-acid presynaptic neuronal protein encoded by the *SNCA* gene [16]. Under physiological conditions it is expressed during neuronal development following the determination of neuronal phenotype and the establishment of synaptic connections [95]. In presence of injuries or altered neuronal plasticity, α-Syn expression decreases, suggesting its important role during synaptic transmission, as demonstrated by Murphy and colleagues by using α-Syn antisense oligonucleotides [96]. On the contrary, α-Syn oligomerization and aggregation seem to be responsible of its enhanced toxicity leading to the degeneration of DAergic neurons in PD [97]. In addition, the secretion of α-Syn have harmful effects on neighboring cells, inducing in turn the aggregation of the endogenous α-Syn, and contributing to spread the disease [98]. α-Syn can be detected in the conditioned medium of cells and extracellular body fluids of PD patients, such as plasma, serum, cerebrospinal fluid (CSF) and saliva [99-101]. However, the low abundance of α-Syn detected in such specimens and the concurrent presence of α-Syn in peripheral sites led to controversial results, making α-Syn an unsuitable biomarker for PD [100, 102].

Importantly, a fraction of extracellular α-Syn is transported inside EVs, thus reviving the possibility to use α-Syn as suitable PD biomarker [97]. The first evidence dates backs to 2005 when Lee and colleagues demonstrated that both RA-differentiated SH-SY5Y neuronal cells and rat primary cortical neurons were able to secrete EVs containing α-Syn [97]. These results were confirmed by Emmanouilidou and colleagues in 2010, who found that the release of toxic α-Syn-EVs was a calcium-dependent process [98]. These data suggested a potential mechanism for an EV-mediated α-Syn spreading between neurons and from neurons to glial cells in the CNS. This cell-to-cell spreading, in turn, increases the release of proinflammatory factors, further exacerbating neurodegeneration [103-105]. Interestingly, in a recent work Guo and colleagues demonstrated that CD11 positive microglia-derived EVs from the CSF of PD and multiple system atrophy (MSA) patients contained higher levels of total α-Syn compared to controls, although no differences were found between PD and MSA patients [106]. Interestingly, when administered to cortical neuron cultures, these vesicles were able to induce severe α-Syn aggregation, thus confirming the ability of EVs, and in particular microglia-derived EVs, to spread α-Syn protein [106]. In this regard, Marie and colleagues in 2015, explained how EVs accelerate exogenous α-Syn aggregation. The researchers demonstrated that the lipid composition of EVs is responsible for α-Syn aggregation, highlighting the important role played specifically by the ganglioside lipids GM2 and GM3 [107].

The presence of α-Syn inside EVs might result from a defective proteolytic activity of lysosomes, whose functions are commonly impaired in PD [108, 109]. For this reason, to avoid its intracellular accumulation, α-Syn may be loaded inside EVs and secreted outside [106, 110]. In this way EVs mediate two events: on one hand they spread α-Syn between cells [111, 112], while on the other, once inside the target cells, EVs are conveyed to lysosomes where α-Syn can be finally degraded [113]. All these findings support the key role played by EVs in PD pathology, not only as possible source of biomarkers, but also as suitable target for therapeutic interventions.

Recently, in 2018, Papadopoulos and colleagues found that the enzyme glucocerebrosidase (GCase) degrades α-Syn to maintain homeostatic levels of α-Syn’s monomeric structure within neuronal cells [114]. More in detail, they found that lowering the activity of GCase, the secretion of α-Syn via EVs was increased [114]. The same year, results from Thomas et al. supported the finding using a Drosophila model [115]. These data support the development of novel therapies to restore GCase activity, and suggest that their efficacy may be evaluated by measuring levels of α-Syn inside EVs [116-118].

### 3.2 α-Syn as EV-transported PD biomarker

To date, a large number of studies analyzed the levels of α-Syn within CNS-derived EVs in plasma samples from PD patients. In all of them α-Syn was higher in PD patients vs. healthy controls (HC). Additionally, an inverse correlation between α-Syn in EVs and GCase enzymatic activity was also found [119-122]. On the other hand, Niu and colleagues in 2020, through a longitudinal study on PD patients, demonstrated a direct correlation between vesicular α-Syn levels and disease severity [123]. Overall, these data support the notion that increased levels of α-Syn, in neuronal EVs isolated from plasma, may be considered a useful biomarker for early PD diagnosis, as well as a predictive marker of motor dysfunction progression in PD.

Following the data obtained using plasma samples, also the levels of α-Syn in serum-derived EVs were explored as a possible reliable PD biomarker. In 2019, Si and colleagues evaluated α-Syn enrichment in CNS-derived EVs from three different clinical groups: (i) HC; (ii) patients with essential tremor (ET), without PD diagnosis; and (iii) early-stage PD patients; this latest group was further divided into two different phenotype-related subgroups: the tremor-dominant (TD) and the non-tremor-dominant (NTD). The investigation demonstrated that the levels of α-Syn in EVs were lower in PD patients compared to ET and HC groups. In particular, within the PD group, α-Syn-EV levels decreased in NTD vs. the TD type [124]. Therefore, measuring α-Syn in serum-derived neuronal EVs may be a useful tool to discriminate between PD, ET patients and HC. This is relevant, considering that ET patients show a risk four-fold higher to develop PD. Also, there is the potential to identify different PD phenotypes, at the early stage of the disease [124].

In contrast with these findings, a recent study from Jiang and colleagues (2020) analyzed the levels of α-Syn in serum-derived neuronal EVs from subjects affected by different NDs, including PD, dementia with Lewy bodies (DLB), rapid eye movement sleep behavioral disorder (RBD), MSA and others. They found a two-fold increase of α-Syn in serum-derived neuronal EVs from prodromal and clinical PD patients when compared to HC, MSA or other NDs. In particular, they found that α-Syn levels were consistently and stably elevated in PD patients at the early or advanced stages, allowing to discriminate clinical PD from controls [125].

More recently, in 2021, Dutta and colleagues analyzed the levels of α-Syn in neuronal (L1CAM-immunoprecipitated) and oligodendroglial (myelin oligodendrocyte glycoprotein-immunoprecipitated) EVs from serum or plasma of PD and MSA patients vs. HC [126]. They found that in both EV types, α-Syn levels significantly increased in the order HC<PD<MSA, in contrast with [125]. Moreover, the ratio between α-Syn in oligodendroglial and neuronal EVs improved the distinction between the two synucleinopathies, with high sensitivity and specificity [126].

Recently, it has been observed that also salivary glands cells release α-Syn in the saliva, either free or associated to EVs. Cao and colleagues, in 2019, found higher levels of oligomeric α-Syn in PD salivary-EVs compared to HC [127]. Also, oligomeric α-Syn over total α-Syn ratio was higher in PD patients [127]. However, in this case, no association with the severity of the disease was found in correlation with oligomeric α-Syn levels in salivary-EVs [127].

The same year, Rani and colleagues demonstrated that neuronal-derived EV levels in saliva were higher in PD patients than in HC [128]. Again, α-Syn in salivary-EVs was increased in PD subjects compared to HC [128]. Although the mechanism by which salivary glands release α-Syn associated to EVs has not been elucidated yet, the presence of neuronal EVs in salivary samples may suggest an interplay between distinct body districts in the α-Syn spreading process, typical of PD.

Studies on CSF-derived EVs provided instead different results. In 2016, Stuendl and colleagues analyzed the levels of α-Syn in CSF-derived EVs from early-stage PD patients vs. age-gender matched HC [129]. Interestingly, they measured lower levels of α-Syn in EVs from early-stage PD subjects compared to HC, although the percentage of α-Syn loaded into EVs was extremely low (2,17%) in both groups. Also, they were able to distinguish between patients with DLB and patients with established PD. CFS samples from DLB patients contained less EVs and lower α-Syn levels compared to both PD patients and HC. Moreover, EVs from DLB and PD groups were able to induce α-Syn oligomerization *in vitro* [129]. Although larger cohort studies are needed to increase the relevance of these observations, α-Syn in CSF-derived EVs may be suggested as a useful biomarker to monitor PD progression. All the findings regarding the specific detection of α-Syn within EVs isolated from different biofluids are reported in Table 1.

**Table 1:** Potential PD biomarkers associated with EVs, and specific levels for each body district.

POTENTIAL PROTEIN BIOMARKERS	EVs SOURCE	LEVELS IN PD PATIENTS	REF.
**α-Syn**	Plasma Saliva CSF Serum	Higher Higher Lower/Higher Lower/Higher	[119-123, 126] [127, 128] [106, 129] [124-126]
**Ser(P)-1292 LRRK2**	Urine	Higher	[135-137]
**DJ-1**	Urine Plasma	Higher Higher	[142, 143] [120]
**APO A1 and J** **Complement C1r**	Plasma	Lower	[144]
**APO D and APO J** **Afamin and Gelsolin** **PEDF**	Serum	Higher	[145]
**PrP^C^**	Plasma	Higher	[146]
**SNAP23** **Calbindin**	Urine	Higher	[149]
**ATP5A, NDUFS, SDHB** **FGF21** **IL-9**	Serum	Lower	[163]
**CRP** **TNF-α** **MIPs**	Serum	Higher	[163]

## 4. Other EV-carried PD protein biomarkers

### 4.1 LRRK2

Leucine-Rich Repeat Kinase 2 (LRRK2) is a large protein kinase composed by several highly conserved functional domains. LRRK2 is mainly found expressed in circulating immune cells, liver and kidney tissues and in brain-specific sub-populations of neuronal cells and astrocytes [130]. This enzyme is encoded by the gene *PARK8*, whose mutations have been related to familial and sporadic late onset PD [131]. LRRK2 is a soluble cytoplasmic protein often found associated with intracellular membranous organelles including mitochondria, lysosomes, endosomes. It is found also in extracellular structures, such as synaptic vesicles and EVs [130, 131]. Although LRRK2 function(s) is still matter of debate, based on its intracellular localization, it may contribute to the biogenesis and/or regulation of trafficking within the endocytic pathway [131, 132]. The mutation G2019S is known to induce an increased autophosphorylation and phosphorylation activity towards Rab substrates [133]. Interestingly, these post-translational modifications do not appear in the cytoplasmic protein or in its free-secreted form, but they are selectively present in LRRK2 inside the EVs [134].

The first evidence of the presence of LRRK2 inside EVs dates back to 2013, when Fraser and colleagues described the possible mechanism by which LRRK2, upon interaction with 14-3-3 protein, is addressed to MVBs and then released in the extracellular space, via EVs [131]. Notably, the authors demonstrated the presence of LRRK2 inside EVs from urine and CFS samples of PD patients. However, LRRK2 levels were not different vs. HC, due to a very high inter-sample variability [131]. Three years later, the same group focused on the LRRK2 Ser(P)-1292 autophosphorylation, which is the result of the specific G2019S mutation, linked with the induction of cellular toxicity. In fact, Ser(P)-1292 level increases following pathogenic mutations of *LRRK2* gene in primary neurons, whereas decreases in response to LRRK2 kinase inhibitors [135]. The authors found that the ratio of Ser(P)-1292 over total LRRK2 was higher in urine-derived EVs from PD subjects carrying the mutation G2019S compared to both non-mutant idiopathic PD patients and HC [135]. Moreover, this ratio was higher also in G2019S subjects with PD compared to healthy G2019S carriers. Consequently, Ser(P)-1292 LRRK2 over total LRRK2 protein ratio might help to predict the risk to develop and manifest the clinical symptoms of PD for G2019S healthy carriers [135]. In addition, the same group measured the levels of Ser(P)-1292 LRRK2 in a larger cohort of idiopathic PD patients vs. HC [136]. First, these levels vary depending on the gender, being higher in men than in women. Second, idiopathic PD patients displayed higher levels of Ser(P)-1292 LRRK2 in urine-derived EVs than controls. Such levels were also positively correlated to several non-motor symptoms and, ultimately, to low quality of life [136]. On the contrary, no significant correlation with the progression in motor impairments was established, presumably because all patients included in the study were treated with pharmacological DAergic therapies [136].

In 2017 Wang and colleagues extended Fraser’s study to a cohort of Norwegian subjects, with or without PD and carrying or not the G2019S *LRRK2* mutation. They isolated EVs from both CSF and urine samples and measured Ser(P)-1292 LRRK2 vs. total LRRK2 protein levels. In urine-derived EVs higher levels of Ser(P)-1292 LRRK2 have been found in G2019S carriers compared to HC, without any significant difference between males and females. However, a significant correlation between G2019S mutation and PD onset has been measured only in males, since asymptomatic subjects had lower levels of Ser(P)-1292 LRRK2. Moreover, they found that Ser(P)-1292 LRRK2 levels were higher in CFS- than in urine-derived EVs, but differences between carriers of G2019S *LRRK2* mutation vs. non-mutant controls were not found [137]. Although the larger number of results confirm the increase of Ser(P)-1292 LRRK2 in urine-derived EVs from PD patients, there are still several aspects that need to be better investigated before considering LRRK2 in EVs as a suitable biomarker for PD.

Additionally, in a recent study, Candelario and colleagues characterized EVs produced by: (i) DAergic neurons derived from induced pluripotent stem cells (iPSC) carrying the *LRRK2* G2019S mutation; (ii) DAergic neurons derived from iPSC of healthy sibling control; and (iii) LRRK2 G2019S edited to correct the mutation [138]. They found that EVs from all cell types were different in size and abundance. In particular, the *LRRK2* G2019S mutation altered the vesicle size. Also, the mutation induced several gene expression changes compared with control and LRRK2 G/S correction. Interestingly, the G/S correction induced the secretion of EVs whose cargo pattern was similar to control ones. Therefore the authors suggested to further analyze the content of EVs to identify potential novel biomarkers for PD [138].

### 4.2 DJ-1

The mutations of *PARK7* gene (encoding for the deglycase DJ-1) have been correlated to familial and sporadic PD. DJ-1 is a small highly conserved dimeric protein mainly expressed in tissues with high energy demand, like testis, the islets of Langerhans and the brain. In the brain, DJ-1 is involved in the regulation of transcription and neuronal protection from oxidative stress. Accordingly, oxidized DJ-1 has been found in post mortem PD patients’ brains [139-141]. The first evidence of the presence of DJ-1 in urine-derived EVs from PD patients have been shown by Ho and colleagues in 2014. As for LRRK2, the authors highlighted a significant increase of DJ-1 in males with PD compared to healthy males [142]. Afterwards, in 2018, when the authors analyzed the levels of oxidized DJ-1 in whole urine samples, without separating the vesicular fraction, they observed a two-fold increase of oxidized DJ-1 in PD patients compared to HC [143].

The same year Zhao and colleagues analyzed DJ-1 plasma levels in PD patients [120]. First, they evaluated the concentration of DJ-1 in the whole plasma by comparing protein levels in PD patients vs. HC. They could not observe any significant difference. However, when they isolated CNS-derived EVs from plasma samples, higher levels of DJ-1 in PD patients were observed compared with HC. In the same samples, also α-Syn was higher in PD patients, confirming the results described in section 3.2. However they did not observe any significant variation between levels of both DJ-1 and α-Syn between early and late stage PD patients [120]. In light of these results, it is not possible to consider DJ-1 as a suitable PD biomarker, yet. Additional studies might provide novel evidences to support its role to reliably monitor PD onset and progression.

### 4.3 Apolipoproteins

The systemic responses induced by PD progression may be monitored through the evaluation of protein changes inside EVs. Therefore, other proteins may be scrutinized as candidates for EV-based PD biomarkers. In particular, Kitamura and colleagues in 2018 isolated EVs from plasma samples of PD patients (two different stages), as well as from healthy subjects. They demonstrated that the levels of Apolipoprotein A1 (APO A1), Apolipoprotein J (APO J) and Complement C1r, which prevent protein aggregation in physiological conditions, decreased in PD patients compared with HC [144]. Similarly, Jiang and collaborators, one year later, observed that the levels of proteins involved in neuroprotection, such as Afamin, Gelsolin, Apolipoprotein D (APO D), APO J and pigmented epithelium-derived factor (PEDF), were higher in serum-derived EVs from PD patients vs. HC [145]. In particular, the levels of these EV-associated proteins gradually increased from mild to severe PD patients, indicating that serum-derived EVs, with their distinct biochemical compositions, might represent a good candidate as source of biomarkers for diagnosis and prognosis of PD.

### 4.4 PrP^C^ and other novel protein candidates

Another finding concerning novel EV-based PD biomarkers has been achieved by Leng and colleagues in 2020 [146]. They measured the levels of the cellular prion protein (PrP^C^) in plasma samples from PD patients with or without cognitive impairments, since this protein has been linked to cognitive decline typical of NDs, such as Alzheimer’s disease (AD) and PD [147]. PrP^C^ is a glycosylphosphatidylinositol-anchored membrane protein mainly located in the CNS, within pre- and post-synaptic sites. Recent findings demonstrated how specific domains of PrP^C^ are used as docking sites for β-sheet rich amyloid proteins, including α-Syn, thus contributing to the transmission of α-Syn to neurons [148]. In their work, the authors found that the levels of PrP^C^ were higher in EVs from PD patients than in HC. Moreover, within the PD group, they observed a significant increase of PrP^C^ in PD patients with cognitive impairments, demonstrating that PrP^C^ in vesicles may be a candidate biomarker to evaluate the severity of the cognitive decline [146].

In 2019 Wang and colleagues [149] performed the proteomic analysis of urine-derived EVs in PD patients vs. HC. They analyzed samples from two independent cohorts of PD patients and HC, all of them free from kidney diseases (which might represent a biased condition) [149]. EV lysates were subjected to mass spectrometry. Among all proteins discovered in urine-EVs, only two, SNAP23 (with 70% sensitivity and 80% specificity), and calbindin (with 76% sensitivity and 71% specificity), were found highly expressed in PD patients vs. HC. The authors suggested that these two proteins, when both expressed, might represent a useful non-invasive biomarker for PD [149].

In 2020 Vacchi and colleagues [150] developed a diagnostic protocol able to differentially diagnose people affected by PD, MSA, atypical parkinsonism with tauopathies (AP tau) vs. HC. Such protocol is based on the analysis of plasma-derived EVs through an innovative flow cytometry approach. First, they found that PD samples were highly enriched in EVs compared to other groups. Moreover, the analysis of EV-associated immune-surface markers revealed that 16 antigens (CD4, CD19, CD45, CD1c, CD2, CD11c, CD31, CD41, CD62, CD146, MCPS, CD25, CD40, CD209 and HLA-ABC) were selectively enriched in PD vs. HC. Interestingly, MSA displayed 12 upregulated surface markers, whereas AP-Tau only 4. PD and MSA shared 11 EV-surface markers, demonstrating that a different immune dysregulation might characterize PD and MSA compared to AP-Tau. Thanks to this novel approach, the authors were able to identify PD patients (97,8% accuracy) and MSA patients (100% accuracy) [150].

As said, the analysis of brain-derived EVs from plasma samples has been also suggested as possible PD biomarker source. Indeed, in 2019 Ohmichi and colleagues [151] measured the abundance of EVs derived from neurons, astrocytes and oligodendrocytes in plasma samples from PD, MSA, progressive supranuclear palsy (PSP) vs. HC. To perform such analysis, the authors developed a specific immunoassay using CD81, a classical small EV marker, in association with donor-cell-type specific markers, such as synaptosome associated protein 25 (SNAP25) for neuron-derived EVs (NDEs), excitatory amino acid transporter 1 (EAAT1) for astrocyte-derived EVs (ADEs) and oligodendrocyte-myelin glycoprotein (OMG) for oligodendrocyte-derived EVs (ODEs). They found that NDEs were significantly higher in PD samples compared to both HC and MSA ones. Considering the clinical severity of PD pathology, they found that all types of brain-derived EVs were higher in advanced PD patients with overt motor symptoms. On the contrary, only NDEs and ODEs (especially NDEs) abundance was significantly higher in mild PD patients compared to HC. Therefore, the authors suggested NDE quantification as the better biomarker to diagnose early stage PD, while ODEs may be used as a surrogate biomarker for monitoring the progression of PD [151]

Overall, these studies suggest that EVs could represent an innovative and useful source of protein biomarkers for both the diagnosis and prognosis of PD. Again, further investigations are needed to verify their specificity and sensibility.

## 5. Mitochondria-derived vesicles as novel biomarker source for PD

Mitochondrial dysfunctions play a pivotal role in PD. This finding was firstly observed during the 80s in people accidentally infused with the neurotoxin MPTP [152]. This molecule, now widely used to reproduce PD in animal models, inhibits the electron transport chain complex I resulting in dysfunctional mitochondrial respiration and increased ROS production, which ultimately lead to DAergic neuron degeneration. As elsewhere reviewed, mutations in PD-linked genes have been correlated with mitochondrial dysfunctions. Additionally, mitochondrial DNA mutations, deletions or rearrangements have been observed in PD patients [153, 154].

Importantly, all mitochondrial dynamics are finely regulated to ensure the organelle homeostasis [155]. Indeed, Mitochondrial Quality Control (MQC) is a four-stage system which include mitochondrial biogenesis, fusion, fission and mitophagy. Impairment in one of these steps leads to mitochondrial dysfunctions commonly observed in aging-related diseases, including PD [156, 157]. Mitophagy is deputed to eliminate the whole damaged mitochondria [158]. On the other hand, MDVs selectively eliminate misfolded proteins or specific parts of damaged mitochondria or, additionally, scavenge ROS [159-161]. MDVs bud off from the mitochondria either to reach the lysosomal compartment for degradation, or to be released outside the cell. For their characteristics and size, MDVs can be considered a specific subtype of EVs [162].

**Figure 2.**
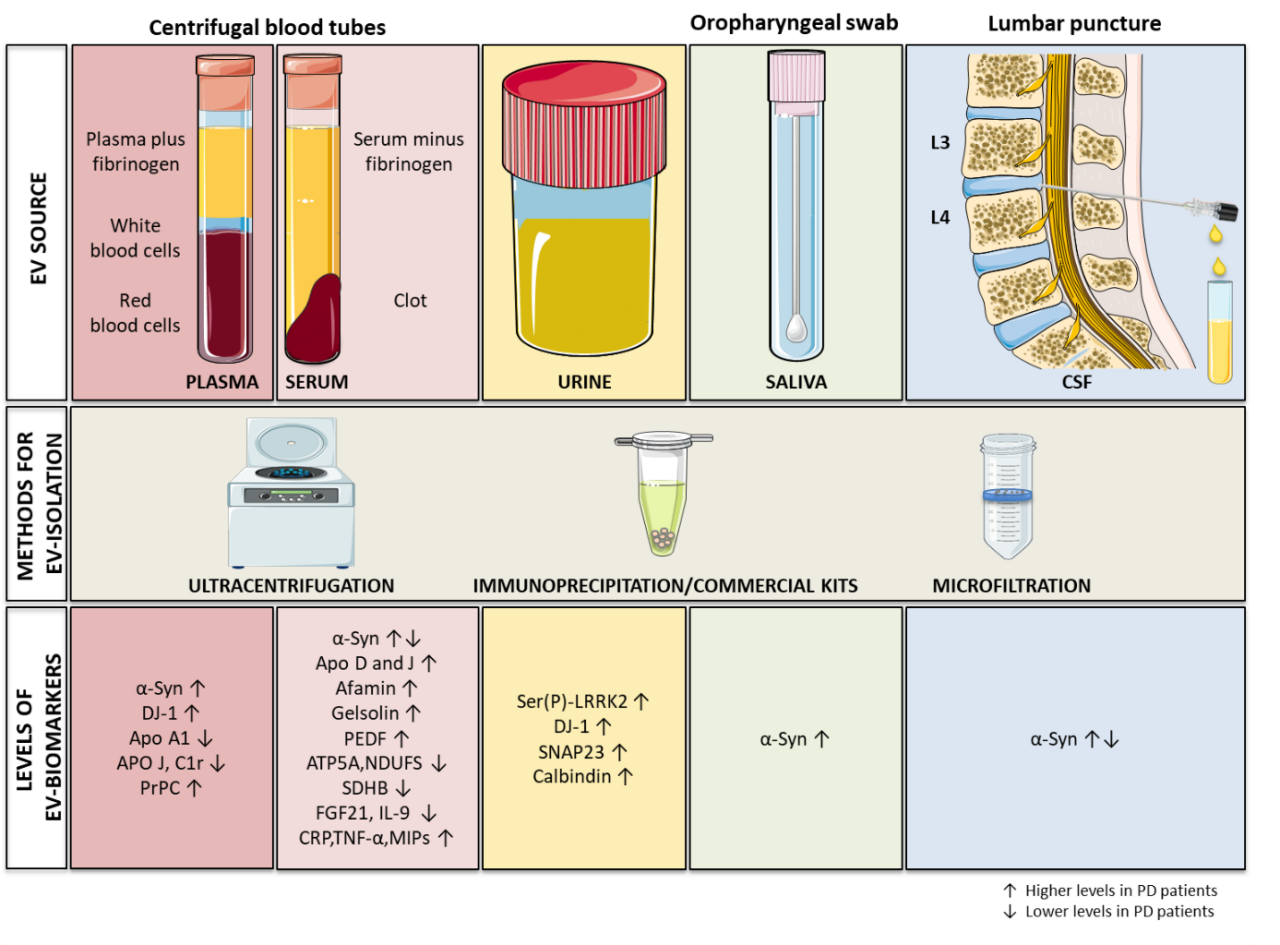
**Schematic representation of EVs isolated from different biological fluids**. EV source (top panels): EVs can be purified starting from different biospecimens, including plasma, serum, urine, saliva, CSF. Methods for EV isolation (middle panel): different approaches have been used to purify EVs, including differential ultracentrifugation, the employment of immunoprecipitation or other commercial kits, microfiltration. EV-biomarkers (bottom panels): published examples of putative PD biomarkers enriched/decreased in EVs (arrows up/down indicate enrichment/decrease).

Recent findings revealed the presence of mitochondrial signatures inside circulating EVs that discriminate between PD patients and HC. Picca and colleagues in 2019 analyzed the protein content of small EVs derived from serum of PD patients vs. controls [163]. To evaluate the presence of MDVs among EVs, the authors analyzed both the classical exosome markers (i.e., CD63, CD9 and CD81), as well as mitochondrial proteins. Moreover, the presence of 27 inflammatory mediators was evaluated within serum samples from both PD patients and HC [163]. From the integrated analysis of small EV content plus inflammatory mediator serum levels, it was possible to accurately discriminate between PD patients and controls. In particular, an increased number of vesicles was observed in PD patients vs. HC, although the levels of CD63 and CD9 were lower in PD patients. Also, mitochondrial proteins from complexes V (ATP5A), I (NDUFS3) and II (SDHB) were found lower in PD serum-derived small EV samples. Moreover, in PD serum samples, lower levels of metabolic modulator Fibroblast growth factor 21 (FGF21) and IL-9 were found, together with a higher concentration of proinflammatory factors (CRP, TNF-α, chemokine macrophage inflammatory proteins (MIPs)) [163]. The authors concluded that the evaluation of mitochondrial proteins and tetraspanins contained in vesicles, combined with the analysis of the inflammatory molecules present in serum, may be useful to discriminate between PD patients and HC [163, 164]. This finding is in line with other researches reporting differential levels of specific proteins within EVs from PD patients vs. controls, indicating that the analysis of EV protein content could serve to identify novel biomarkers for PD. In Table 1 and Figure 2 we summarized all the novel EV-based protein biomarker candidates for PD.

## 6. miRNAs carried by EVs as PD biomarkers

**Figure 3.**
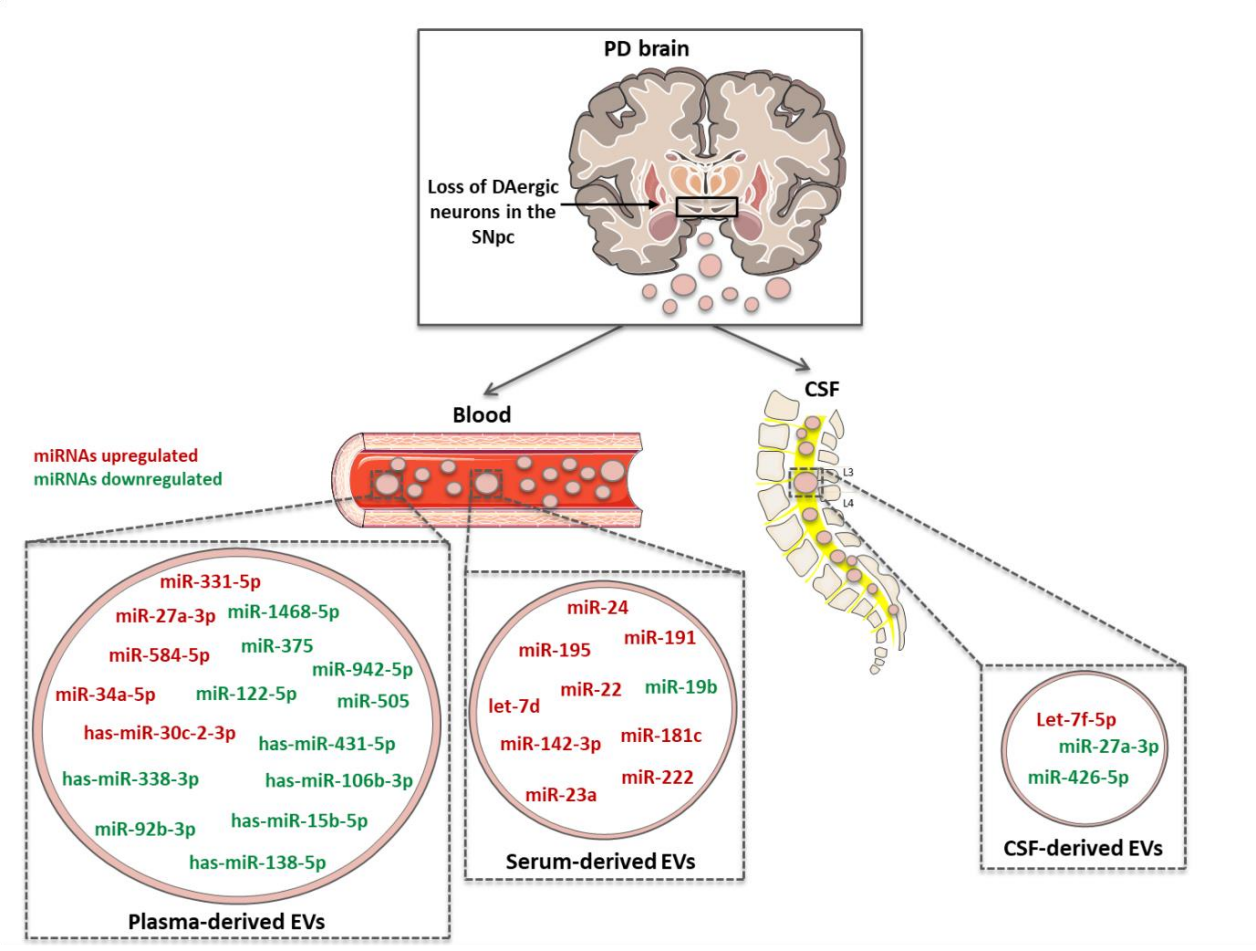
**Schematic representation of CNS-derived EVs from PD patients, carrying dysregulated miRNAs**. CNS-derived EVs, recovered from CSF, serum or plasma, contain miRNAs whose levels has been found higher (red) or lower (green) compared to controls. These specific signatures may be useful as possible biomarkers for PD.

Micro-RNAs (miRNAs) are small non-coding RNAs that repress gene expression via the interaction with target mRNAs [165]. They play an important role both in physiological and pathological conditions, as recently reviewed in PD from our group [63]. Herein, we collected all findings from 2017 to present, focusing on miRNAs specifically carried by EVs, and their role as possible biomarkers for PD (Fig. 3). In 2017, Cao and colleagues characterized the profile of 24 EV-miRNAs, previously identified as potential biomarkers for PD in serum or plasma samples [166]. They isolated EVs from serum of 109 PD patients and 40 HC. Among the 24 miRNAs, only 3 miRNAs were found consistently present in both PD cases and controls. In particular, miR-24 and miR-195 were significantly higher in serum-EVs from PD patients, whereas miR-19b was significantly lower compared to HC [166]. The same year, Aghili and colleagues developed a nano-biosensor able to detect early PD by specific identification and quantification of miR-195 in serum samples [167, 168].

One year later, in 2018, Dos Santos and colleagues, investigated EV-miRNAs enriched in CSF samples from early stage PD patients [169]. Through the use of a machine learning approach, they predicted that early-stage PD patients have high levels of let-7f-5p and low levels of miR-27a-3p and miR-426-5p, whereas controls should have high levels of miR-125a-5p and low levels of miR-151a-3p. These miRNAs, although never proposed as PD biomarkers, are involved in several biological pathways linked with PD. Interestingly, the combination of miRNA analysis with specific protein biomarkers, such as α-Syn, allowed the identification of other miRNAs correlated with α-Syn levels in CSF. In particular, early stage PD patients should express low levels of both α-Syn and miR-22-3p, as well as high levels of miR-10b-5p and miR-151a-3p [169]. Although this approach bears a great predictive power, further sequencing-based validation in patients is needed [169].

The same year, Yao and collaborators compared miRNA levels in plasma and plasma-derived EVs from PD patients vs. HC [170]. They found no difference in plasma miRNAs between PD and controls. On the other hand, the analysis of miRNA levels in plasma-derived EVs identified miR-331-5p as significantly enriched in PD patients compared to controls, whereas miR-505 was significantly lower [170]. Indeed, when they compared the levels of these two miRNAs in EVs and plasma, they confirmed the enrichment of miR-331-5p in EVs, whereas miR-505 was enriched in plasma samples [170].

A known risk factor for PD and PD-like pathologies is the exposure to neurotoxicants [171]. For example, the chronic exposure to environmental manganese (Mn) determines the insurgence of a neurological syndrome with motor dysfunctions slightly similar to PD [172]. In this regard, in 2018, Harischandra and colleagues developed an *in vitro* model of MN9D DAergic cells expressing a GFP-tagged α-Syn and treated with Mn [173]. They found that Mn exposure induced a significant increase of EV secretion. Also, Mn exposure altered the small RNA profile of EVs secreted by cells with GFP-tagged α-Syn. In details, they found 12 miRNAs (miR-210-5p, miR-128-1-5p, miR-505, miR-325-5p, miR-16-5p, miR-1306-5p, miR-669b-5p, miR-125b-5p, miR-450b-3p, miR-24-2-5p, miR-6516-3p and miR-1291) enriched in Mn-exposed α-Syn cell-derived EVs compared to non-exposed cells. These miRNAs regulate multiple relevant biological pathways (such as mitochondrial functions, autophagy, inflammation and protein aggregation) and can be suggested as possible biomarkers to be assessed within *in vivo* PD studies [173].

In 2019, Barbagallo and colleagues [174] investigated the expression of 23 miRNAs contained in serum-derived EV isolated from a cohort of 139 individuals including patients affected by AD, PD, vascular dementia (VD), vascular parkinsonism (VP) and HC. Regarding PD patients, the group identified a set of 8 miRNAs (let-7d, miR-22*, miR-23a, miR-24, miR-142-3p, miR-181c, miR-191 and miR-222) differentially expressed in serum-derived EVs from PD patients vs. HC. Also, 3 miRNAs (miR-23a, miR29a, and miR181c) were differentially expressed in PD compared with VP specimens. Additionally, the authors identified 13 miRNAs (let-7d, miR-1, miR-9, miR10b*, miR-15b, miR-19b, miR-22*, miR-23a, miR-24, miR-29a, miR-29b, miR-29c, miR-34b, miR-125b, miR130b, miR-137, miR-142, miR-148b, miR-181c, miR191, miR-222, miR-324, and miR-505) selectively overexpressed in all ND serum-derived EVs compared to HC. Moreover, upon correlation of specific miRNA levels with the age of patients, the authors observed a significantly positive correlation with age for let-7d, miR-24, miR-29a and miR-222 in PD group, for miR-130b in VP group and for miR-24 and miR-130b in PD and VP groups when clustered together. No statistically significance was found upon correlation of miRNA expression and gender of the subjects [174].

A recent study from Xie et al. in 2020 [175], measured the enrichment of specific miRNAs in plasma-derived EVs from 30 PD patients and 30 HC. They found that plasma-derived EVs from PD patients carried higher levels of miR-30c-2-3p compared to controls. On the contrary, miR-15b-5p, miR-138-5p, miR-338-3p, miR-106b-3p and miR-431-5p levels were lower in PD than in HC. Interestingly, GO and KEGG pathway analyses revealed that all dysregulated miRNAs (except miR-106b-3p), targeted genes involved in DAergic synapses formation, neurogenesis and neuron protection guidance, further supporting their critical role in PD progression [175].

The same year, Nie and colleagues analyzed miRNA expression profile in plasma-derived EVs from patients with AD and PD [176]. The study involved 34 HC, 5 AD patients and 7 PD patients. RNA-seq data revealed that miR-27a-3p and miR-584-5p were higher in both AD and PD patients vs. HC. On the contrary, 5 miRNAs (miR-942-5p, miR-92b-3p, miR-375, miR-122-5p and miR-1468-5p) were downregulated in both disease samples compared to controls. Moreover, the comparison between AD and PD samples revealed that among the 23 low-level miRNAs in AD, 4 were also low represented in PD, while only one (i.e., the let-7e-5p) was enriched in PD. Therefore let-7e-5p has been suggested as a possible biomarker able to selectively discriminate between AD and PD patients. However, considering the small sample size and some differences with previously published data, the authors suggested to further validate the candidate miRNA-biomarkers in larger longitudinal studies [176].

More recently, in 2021, Grossi and colleagues investigated the role of miR-34a-5p, which is involved in other pathologies of the CNS [177], as a potential biomarker for PD [178]. They analyzed EVs derived from plasma samples of PD patients and age-matched controls via qPCR. To selectively isolate different EV subtypes, the authors used a standard protocol based on differential ultracentrifugation steps. In this way they separated large EVs (800 x g), medium EVs (16,000 x g), small EVs (100,000 x g) and “pure” small EVs (upon subsequent sucrose gradient separation). First, they evaluated the total miRNA (ng) content, which was found higher in pure EVs compared to the other EV types. Then, they focused on the levels of miR-34a-5p in pure small EVs from all subjects’ specimens. Interestingly, the analysis revealed a significant increase of this miRNA in patients within 5 years from PD onset, compared to HC [178].

All these findings suggest that the research in the field is growing at fast pace. The availability of novel and more accurate protocols, as well as research tools to isolate EVs from body fluids, together with improved multi-omics approaches, will potentially ensure more precise identification of novel miRNA biomarkers for diagnosis/monitoring of PD insurgence/development.

## 7. Monitoring PD therapy outcomes through EVs

As previously mentioned, EV-based biomarkers can be used also to evaluate the efficacy of available and novel therapeutic approaches to treat PD. In 2016, Luo and colleagues investigated the effects of pramipexole, an aminobenzothiazole non-ergoline DA agonist, administered to PD patients at different dosages. The authors evaluated the motor functions before and after the therapy, proving that pramipexole produced significant functional improvements. Thus, they demonstrated pramipexole efficacy in 52 over a total of 65 PD patients. Additionally, they evaluated α-Syn levels contained in serum-derived EVs and they observed a significant correlation between α-Syn levels and pramipexole therapeutic efficacy. People who positively responded to therapy showed significantly lower levels of α-Syn carried in serum-derived EVs compared to non-responders. These findings suggest that the efficacy of the pramipexole treatment may be monitored through the use of a non-invasive analysis based on the measurement of α-Syn levels within serum-derived EVs [179].

In line with this study, Athauda and colleagues in 2019 demonstrated the potential use of CNS-derived EVs from serum samples of PD patients as source of biomarkers to monitor 60 weeks treatment with exenatide [180]. Exenatide is a glucagon-like peptide 1 agonist used to cure type II diabetes and repurposed for PD treatment. The authors observed that exenatide activated the insulin signaling pathway through phosphorylation of insulin receptor substrate 1 (IRS-1 p-Tyr) in neural-derived EVs isolated from serum. As a result, the downstream activation of Akt and mTOR signaling pathways was found higher in PD treated patients compared to placebo-assigned controls. This signaling activation was correlated with improved motor functions in exenatide-treated PD patients. The authors suggested a direct correlation between IRS-1 phosphorylation levels in serum-derived neuronal-EVs and the mTOR signaling activation, coupled with the motor function amelioration. These results pinpoint that exenatide acts via the activation of brain insulin signaling pathway. Its effectiveness may be monitored through the analysis of CNS-derived EVs isolated from patients’ serum and carrying relevant biomarkers (such as IRS-1). Therefore, IRS-1 in EVs may be suggested as follow-up biomarker to monitor the efficacy of therapies targeting neuronal pathways [180].

Following the evaluation of Ser(P)-1292 LRRK2 expression in CSF-derived EVs (described in section 4.1), which supported the role of auto-phosphorylated LRRK2 as potential PD biomarker [137], Wang et al. in 2020 further characterized Ser(P)-1292 LRRK2 as pharmacodynamic marker of LRRK2 drug-inhibition in macaques [181]. In fact, quantitative measure of LRRK2 inhibition, especially in the brain, may be critical in the development of successful LRRK2-targeting therapeutics. Two brain-penetrant and selective LRRK2 inhibitors (PFE-360 and MLi2 small-molecules) were orally administered to the animals. The authors observed that, upon selective LRRK2 inhibition, the LRRK2-substrate pT73-Rab10 was found reduced in urine, while auto-phosphorylated pS1292-LRRK2 protein was found reduced in CSF. In addition, both total LRRK2 and Ser(P)-1292 LRRK2 levels were found diminished in CSF-derived EVs [181]. Overall, these results demonstrated the utility of combining biofluid analyses in both vesicular and non-vesicular components to quantify LRRK2 inhibition in the whole body [181].

## 8. Conclusions

PD represents the second most common progressive ND, characterized by the loss of DAergic neurons of the SN. A central role in the complex etiology of the disease is played by genetic and environmental factors, such as aging, inflammation and oxidative stress. In particular, the aging process impairs the nigrostriatal DAergic system at neurochemical, morphological and behavioral levels, and inhibits DAergic neuroplasticity and repair, including adult neurogenesis. The age-dependent loss of anti-oxidant, anti-inflammatory and neuroprotective glial functions further increase DAergic vulnerability to a host of harmful factors, with detrimental consequences for DAergic neuron health.

The urgence of identifying novel biomarkers for PD depends on the fact that the first motor signs appear decades after the initial insult. At that time, a significant proportion of SN neurons are already lost, making any type of therapeutic intervention ineffective. New trials aiming to cure the disease, or at least to halt its progression, have not been successful, yet. To this end, considerable efforts are devoted to the identification and screening of valuable clinical PD biomarkers, to reveal early aspects of disease pathogenesis and eventually to develop diagnostic methods to prevent and treat PD.

We herein propose EVs as novel sources of biomarkers in PD. CNS-derived EVs may be found in almost all body fluids, such as CSF, serum, plasma, urine and saliva. In PD, EVs have been initially identified as the carrier of pathological α-Syn, which is spread within and outside the brain. In line with this finding, α-Syn levels have been analyzed to discriminate PD patients from healthy controls. With all the limitations deriving from the different protocols used to isolate EVs, α-Syn in EVs stands as a possible biomarker for PD. Other potential biomarker candidates have been found enriched in PD-derived EVs compared to controls. For instance, higher levels of phosphorylated Ser(P)-1292 LRRK2. The same trend was observed for DJ-1. Moreover, other proteins (such as PrP^C^, APO A1, APO J etc.) have been scrutinized as EV-based biomarkers for PD. Again, further investigation is needed to increase the number of clinical samples and validate them as PD biomarkers.

EVs may also transport other pivotal regulators of PD-related pathways, such as miRNAs, which *per se* represent interesting molecules for PD diagnosis and monitoring. In addition, MDVs are currently explored as innovative source of vesicle-carried PD biomarkers.

Importantly, the analysis of changes in protein/miRNA content, within a specific EV population, both in untreated PD patients and upon pharmacological treatment, represents another important step forward in the EV-based research. This would represent a non-invasive strategy to monitor the efficacy of a specific therapy and possibly predict the outcomes of PD.

Although some of the studies remain controversial, they demonstrate how technological and methodological progresses are helping to ameliorate both PD diagnosis and prognosis. Ultimately, in the *Era* of personalized medicine, this effort may be posing the bases to identify at risk population and to gather a positive impact on the quality of life of PD patients.

## 9. Future perspectives

EVs play key roles in cell-to-cell communication, making them potential candidates to sense the changing microenvironment and to quickly respond to initial threats/stressors with specific molecular signatures. This may enable the “anticipation” of the clinical phase of PD, as well as inform about its progression and/or the response to therapy. As described above, EVs possess several features that make them “ideal” molecular carriers (i.e., they transport a heterogeneous group of molecules, they can cross the BBB, they are stable in biofluids). A major challenge for the field is represented by the fact that EVs are released by many cell types both in the brain and the periphery. Thus, in the nearer future, it will become essential to develop and to standardize procedures which allow to tag the exact EV-cellular origin [182].

Presently, about 30 clinical trials registered at *clinicaltrials.gov* are exploring the analysis of EVs as source of specific disease-related biomarkers (including cancer, obesity, thrombosis, SARS-CoV2 infection, aging and NDs). Focusing on PD, two clinical studies are currently ongoing, both sponsored by the Michael J. Fox Foundation for Parkinson's Research. The first one (NCT03775447, Fox BioNet Project ExtraCellular Vesicles ECV-003), is a recently concluded case-control study which enrolled 38 subjects with the goal of optimizing pre-analytical EV isolation protocols for increasing the detection of LRRK2 activity in human CSF. The second (NCT04603326, Fox BioNet Project ECV-004) is currently enrolling up to 140 individuals (PD patients and healthy controls) with the goal of identifying reliable markers of LRRK2 activity in both CSF and EVs-CSF.

The possibility to routinely investigate EVs mainly depends on the techniques used to process the samples and, importantly, to isolate EVs [183]. For example, for plasma-derived EVs, a critical role is played by the specific anticoagulant used. It has been shown that acid citrate dextrose better decreases the presence of platelet microparticles, compared to other anticoagulants, whose presence may compromise the results [183]. Interestingly, the protein composition of EVs isolated from plasma obtained with different anticoagulants, as well as from serum, demonstrated a variability linked with the method used [183]. This could be an issue, given that the protein presence in EVs might be modified depending on the specific plasma or serum isolation protocol.

At present, several EV-isolation techniques are under study as reliable alternatives to ultracentrifuge-based methods, which are time-consuming and very expensive. Other possible user friendly approaches are represented by antibody-coated micro-surfaces (e.g. beads or flat surfaces), precipitating agents, ultra-filtrating membranes, as well as columns for size exclusion chromatography (already reviewed in [184]). However, each of these approaches show pros and cons. In particular, the different results in terms of yield of recovered EVs, miRNA and protein enrichment and content [184]. In terms of standardization and automatization, microfluidic devices are among the most explored isolation tools [185]. Overall, they may represent an easy-to-use method for the robust isolation/purification of EVs from biological fluids. In the future, such technical improvement will sensibly speed-up the validation of novel diagnostic and prognostic biomarkers, as well as therapeutic drug applications of EVs.
